# Improving Undernutrition with Microalgae

**DOI:** 10.3390/nu16183223

**Published:** 2024-09-23

**Authors:** Sunil K. Panchal, Kirsten Heimann, Lindsay Brown

**Affiliations:** 1 School of Science, Western Sydney University, Richmond, NSW 2753, Australia; s.panchal@westernsydney.edu.au; 2 College of Medicine and Public Health, Flinders University, Health Science Building, Building 4, Registry Road, Bedford Park, Adelaide, SA 5042, Australia; kirsten.heimann@flinders.edu.au; 3 School of Pharmacy and Medical Sciences, Griffith University, Gold Coast, QLD 4222, Australia

**Keywords:** microalgae, nutrition, omega-3 fatty acids, protein, functional foods

## Abstract

Undernutrition is an important global health problem, especially in children and older adults. Both reversal of maternal and child undernutrition and heathy ageing have become United Nations-supported global initiatives, leading to increased attention to nutritional interventions targeting undernutrition. One feasible option is microalgae, the precursor of all terrestrial plants. Most commercially farmed microalgae are photosynthetic single-celled organisms producing organic carbon compounds and oxygen. This review will discuss commercial opportunities to grow microalgae. Microalgae produce lipids (including omega-3 fatty acids), proteins, carbohydrates, pigments and micronutrients and so can provide a suitable and underutilised alternative for addressing undernutrition. The health benefits of nutrients derived from microalgae have been identified, and thus they are suitable candidates for addressing nutritional issues globally. This review will discuss the potential benefits of microalgae-derived nutrients and opportunities for microalgae to be converted into food products. The advantages of microalgae cultivation include that it does not need arable land or pesticides. Additionally, most species of microalgae are still unexplored, presenting options for further development. Further, the usefulness of microalgae for other purposes such as bioremediation and biofuels will increase the knowledge of these microorganisms, allowing the development of more efficient production of these microalgae as nutritional interventions.

## 1. Introduction

Human nutrition encompasses the supply of nutrients in food to support human life and health. Nutritional deficiency remains a chronic health problem throughout the world. The Global Burden of Disease project reported that the major risk factor for disability-adjusted life years (DALYs) globally was maternal and child undernutrition, accounting for 295 million DALYs or 11.6% of the total DALYs in 2019 [1]. The WHO and UNICEF estimate that globally, 149.2 million or 22.3% of children under the age of 5 years are stunted (too short for their age) and 45.4 million are wasted (underweight for their height). Around 45% of the around 4.5 million deaths among children under 5 years of age are linked to undernutrition [2]. Further, the WHO advises that better nutrition improves infant, child and maternal health, lowers the risk of non-communicable diseases, improves productivity and creates opportunities to break cycles of poverty and hunger [3].

The problem of undernutrition is not restricted to children. Undernutrition is a significant health issue in older adults, resulting from a combination of lifestyle, social and physiological factors [4]. Undernutrition in older adults affects their health and quality of life by increasing the risks of chronic disease development, weight loss, muscle mass loss, diminished physical and mental functioning and impaired clinical outcomes [4,5]. Nutrients (especially protein) and energy are the common factors that are reduced in older adult-associated malnutrition [4,5]. Clinical guidelines for the management of undernutrition in older adults include assessment of undernutrition and development of nutritional support [6].

This review will discuss the role that microalgae could play in alleviating chronic undernutrition throughout the world. Further, it will highlight recent advances in microalgal cultivation which can produce increased amounts of microalgal biomass and more products. It will further discuss opportunities to develop food products from microalgae that will allow viable incorporation of microalgae into different diets. Additionally, we will briefly highlight other uses of microalgal biomass in industry for alternative commercial opportunities.

## 2. Microalgae in the Environment

Phytoplankton are microscopic organisms living in aquatic environments and include cyanobacteria, diatoms, dinoflagellates, green microalgae such as *Mamiellophyceae* species and red microalgae such as *Porphyridium* species [7,8,9]. The evolutionary origins of marine algae and the environmental success of green algae leading to all terrestrial plants, together with their economic importance and their role in human life, have been succinctly outlined [10]. Single-celled photosynthetic prokaryotes appeared early in the Earth’s development, probably around 2.7–3.5 billion years ago, including the oldest fossil evidence of cyanobacteria dated to about 2.15 billion years ago [11]. Eukaryotes such as microalgae appeared around 1–1.5 billion years after the emergence of prokaryotes. Photosynthetic phytoplankton use photosynthesis with chlorophyll *a* to consume carbon dioxide and release oxygen. Photosynthesis by cyanobacteria considerably predates the first photosynthesis in rainforests of giant ferns, which appeared about 390 million years ago [12], and the current rainforests of angiosperms, which appeared about 60 million years ago [13]. The role of cyanobacteria in the Great Oxidation Event, when oxygen became a major component of our atmosphere around 2.1–2.4 billion years ago, is still debated [11,14]. Marine phytoplankton currently produce more than 45% of the photosynthetic net primary production on Earth [15]. As an example, the marine bacteria *Prochlorococcus* produces about 20% of the world’s oxygen, which may be decreased by plastic leachates [16]. This oxygen production is greater than that of all the tropical rainforests [17].

Most algae are described as eukaryotic organisms that photosynthesise, that is, they use light energy to convert inorganic carbon into organic matter, but they lack the specialised multicellular reproductive structures of plants, true roots, stems or leaves; prokaryotic cyanobacteria such as blue-green algae or Cyanophyta are sometimes included because of their similar physiology and ecology [18]. Algae can be broadly divided into the multicellular macroalgae, including seaweeds, and the unicellular microalgae. Macroalgae have been part of the human diet for many generations, especially in east Asia; their role in healthy ageing has been recently reviewed [19]. Microalgae are a diverse family with many as yet uncharacterised, with some research estimating that there are 72,500 microalgal species [20]. This is about twice as many as the number of species of terrestrial plants and so provides an enormous natural resource. Microalgae grow rapidly with a theoretical maximum net biomass accumulation of 77 ± 5 g dry cell weight/m^2^/day or 280-ton dry cell weight/ha/year; small-scale green microalgal productivity has been estimated at 27–54% with a solar-to-product energy conversion efficiency of 3% [21].

The production of biomass from the marine environment (including both animals, especially fish and algae) is a major industry around the world, producing 214 million tonnes in 2020 to provide an average annual consumption of 20.4 kg/person [22]. However, algae are only a small part of commercial marine biomass with a global production of about 36 million tonnes in 2020, with most of this being macroalgae; the commercial production of microalgae in 2019 was reported to be 56,465 tonnes, or less than 0.2% of global production of marine biomass [22]. The FAO reports these data as incomplete due to non-disclosure by countries such as the USA and Australia [22]. In 2019, China produced 97.16% of microalgae, with *Arthrospira* (commonly known as *Spirulina*) comprising 96.56% of global microalgae production [23].

Microalgae are not restricted to marine environments but also occur in freshwater sources such as rivers and reservoirs [24]. Freshwater microalgae contain a wide range of compounds with nutritional properties, including lipids, carbohydrates, proteins and vitamins, and therefore should also be considered potential food sources [25]. Further, multifunctional microalgae in soil improve fertility and plant growth, remove pollutants and control crop diseases [26]. In addition, green microalgae from extreme environments contain anti-bacterial compounds that may have activity against human pathogens [27]. Microalgal biosynthetic pathways for fatty acids and pigments have been summarised [28,29], while the synthesis of amino acids and carbohydrates follows the same pathways across kingdoms.

## 3. Nutritional Components of Microalgae

**Figure 1:**
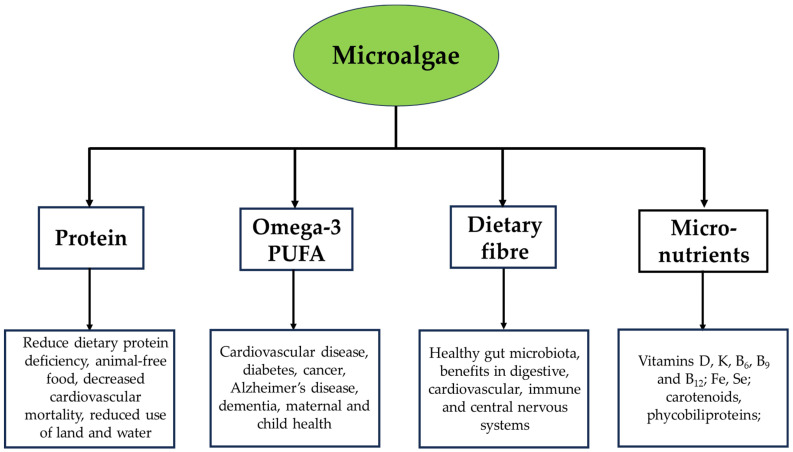
Major components of microalgae and their potential benefits.

This section will highlight the components of microalgae and their potential benefits. The biochemical composition of microalgae varies between species (Table 1). The highest protein contents are typically found in species of *Chlorella* and *Arthrospira*; high carbohydrate concentrations are found in red microalgae such as *Porphyridium,* while highest lipid contents, particularly for the long-chain omega-3 fatty acids eicosapentaenoic acid (EPA) and docosahexaenoic acid (DHA), are found in *Nannochloropsis* and thraustochytrids such as *Schizochytrium*, respectively. Thus, microalgae can be considered potential sources of lipids, carbohydrates and proteins in human and animal diets (Figure 1). Further, microalgae are relevant as sources of pigments and micronutrients (vitamins and minerals), which could be included in foods and nutraceuticals for promoting human health [30,31] (Figure 1). Table 2 highlights the lipid composition of microalgae including the content of EPA and DHA. Table 3 estimates the global production of microalgae that would be needed to alleviate global deficiencies in omega-3 polyunsaturated fatty acids (PUFA), dietary fibre and protein. Table 4 summarises their benefits according to some clinical studies of animals and microalgae, either as whole biomass, lipid, carbohydrate, pigment, mineral or water/ethanol extract.

Defining the relevance of including microalgal-derived nutrients in overcoming undernutrition requires an examination of the current sources of these nutrients, such as cereals and meat, to determine whether these sources can be readily increased to provide adequate supplies for the world’s population. If the current sources cannot be realistically and quickly expanded to meet demands, then developing alternative sources such as microalgae must become a priority.

### 3.1. Lipids

Fats, a major source of energy in the human diet, consist of fatty acids which are carboxylic acids with an aliphatic chain, which can be saturated or unsaturated. However, generalising fatty acids by their chemistry does not predict physiological responses [53]. Most dietary fat sources contain both saturated and unsaturated C18-fatty acids producing differing metabolic and cardiovascular responses. In a rat model of diet-induced metabolic syndrome, physiological responses to unsaturated C18-fatty acids (monounsaturated oleic acid, omega-6 linoleic acid and omega-3 α-linolenic acid (ALA)) included decreased *trans*-fatty acids in the heart, liver and skeletal muscle, and increased left ventricular dilatation and plasma glucose with oleic and linoleic acids; however, responses also included improved heart structure and function and glucose tolerance with ALA, decreased abdominal fat with EPA and DHA, and decreased plasma triglycerides and cardiac fibrosis [54]. In humans, MUFA showed anti-inflammatory responses which could contribute to the benefits of the Mediterranean diet, while SFA seemed to be pro-inflammatory, making dietary fats both pro- and anti-inflammatory [55]. An extensive range of pharmacological actions have been attributed to ALA, including anti-inflammatory, antioxidant, anti-obesity and neuroprotective effects [56]. Two long-chain PUFA, linoleic acid and ALA, are essential fatty acids for humans as they cannot be synthesised in the body. Humans can inefficiently convert ALA to the longer-chain EPA and DHA, suggesting that additional dietary intake is necessary for optimal health. The major current source of EPA and DHA is fish oil; reviews of epidemiological studies and clinical trials with fish oil, especially for cardiovascular disease, have been continually published for over 30 years [57,58,59]. The wide variety of human diseases with reported health benefits from the combination of EPA and DHA in fish oil include cardiovascular disease, diabetes, cancer, Alzheimer’s disease, dementia, depression, visual and neurological development, and maternal and child health [60]. Other assessments have been less optimistic, with a Cochrane assessment reporting that increasing long-chain omega-3 PUFA slightly reduces risk of coronary heart disease and reduces serum triglycerides with little to no effect on coronary and all-cause mortality [61]. Independent responses to EPA and DHA, and possibly docosapentaenoic acid (DPA), are potential reasons for differences in pharmacological responses. For example, a clinical trial with an EPA-only formulation lowered cardiovascular events by 25% in patients with established cardiovascular disease or diabetes, while trials with DHA-containing high-dose omega-3 PUFA formulations have not shown these benefits of EPA alone [62]. Possible reasons include ready incorporation of EPA in plaques and differences in their rates of lipid oxidation, inflammatory biomarkers and endothelial function [62]. However, DHA is at much higher concentrations in the brain than EPA, so the focus in studies on neuroprotection has been on DHA [63,64].

From Table 3, it is difficult to see how current fish oil production can be sufficiently increased to meet global recommendations for health intake. One potential option is to selectively increase production and consumption of oily fish. For Australians, the Heart Foundation recommends including 2–3 servings of fish (including oily fish) each week to provide around 250–500 mg marine-sourced EPA and DHA per day [65]. Surveys have shown that oily fish consumption is markedly lower than guidelines; for example, it was between 3.4 and 19.1 g per day in the UK population as of 2016/17, with only 15.6% of young adults meeting recommendations of at least 20 g oily fish per day [66]. Increasing consumption of oily fish by 10 g/day for the world’s population would require around 25 million tonnes/year of oily fish, yet commercial tuna production was around 5.8 million tonnes in 2018, and Atlantic salmon production in 2022 was less than 2.9 million tonnes [67]. Thus, the large gaps in intake of omega-3 PUFA remain the premise for the development of alternative models of production and delivery, including fortification of food, bioengineered plants, microencapsulation to increase patient acceptance and PUFA bioavailability, as well as increased production from plants and yeasts including microalgae [66].

Although fish store omega-3 PUFA, these fatty acids are synthesised by plants and yeasts, including micro- and macroalgae, and by genetic modification of plants, microalgae, bacteria, fungi and yeasts [68,69]. Some plants synthesise shorter-chain omega-3 PUFA such as ALA or stearidonic acid (SDA; 18:4n − 3). SDA is efficiently converted to EPA, unlike ALA, as SDA bypasses the rate-limiting step in the conversion of ALA to EPA. SDA occurs in concentrations of 20–36% in the oils from seeds of flowering plants such as *Echium plantagineum* L., *Ribes nigrum* L. and *Cannabis sativa* L.; SDA has shown health benefits in inflammation, cardiovascular and metabolic diseases, and cancer possibly after conversion to omega-3 fatty acids [70]. DPA is the intermediate step between EPA and DHA. Intake of omega-3 fatty acids, ALA, eicosatetraenoic acid and DPA but not EPA or DHA has been associated with reduced risk of cardiovascular disease in 31,184 adults in NHANES 1999–2018 [71], but no clinical trials of DPA have been found in the literature. The Heart Foundation recommends the inclusion of 1 g of plant-sourced ALA per day, for example, from walnuts, linseed, chia, canola or soybean [65]. The definition of the individual therapeutic effects of ALA (rather than its metabolites, EPA and DHA) in cancer, insulin resistance and cardiovascular disease was identified as an important research goal as ALA is the major PUFA in westernised diets [72]. Later research showed that ALA produced similar cardiovascular and metabolic responses to EPA and DHA with limited accumulation of EPA or DHA in a rat model of diet-induced metabolic syndrome [73]. Further, dietary ALA may reduce the risk of cardiovascular disease through anti-hypertensive, anti-atherosclerotic and cardioprotective effects [74].

However, as the primary producers of EPA and DHA in the marine food chain, microalgae can serve as a sustainable option for an efficient large-scale commercial cultivation system. With short harvesting time, microalgae can address global demand for the production of EPA and DHA [75]. Some microalgae can produce 22–45% EPA + DHA of the total lipid fraction [75,76]. To obtain 1000 mg EPA + DHA, ~110 g of fresh microalgae or ~11 g dry microalgae would be required, considering the 90% moisture content of microalgae and the 30% EPA + DHA content of the lipid fraction. To provide the 294,000 tonnes needed to bring 66.8% of the world’s population to the recommended intake of 250 mg EPA + DHA/day, about 3.2 million tonnes of dry-weight microalgae would be required each year (Table 3). Similar calculations can be performed to estimate the microalgae production needed to reverse dietary fibre and protein undernutrition (Table 3). While these numbers are very large compared to current estimates of microalgae production, they are less than 1/10th of the WHO’s estimation of the total production of marine biomass in 2020, which was around 36 million tonnes [22].

The ratio of EPA to DHA in marine phytoplankton varies considerably (Table 2) with highly class-specific PUFA production including EPA/DHA ratios from 0.3 in *Dinophyta* to 9.2 in *Diatoma* [77]. Microalgae such as *Nannochloropsis* sp. produce almost exclusively EPA rather than DHA as the omega-3 PUFA [78]. In contrast, the production of DHA is achieved by heterotrophic protists including the thraustochytrids such as *Thraustochytrium*, *Aurantiochytrium* and *Schizochytrium* as they contain the relevant biochemical pathways, including the elongase-desaturase pathways [79]. Further, strategies for improving microalgal production of omega-3 PUFA have now been suggested and investigated, including genetic engineering, environmental conditions, symbiotic bacteria and fungi, and nutritional factors [69,70,80]. Further, transgenic plants such as *Camelina sativa* L. synthesise EPA and DHA at concentrations similar to fish oil following reconstitution of the biosynthetic pathway [81]. There are no reported microbial sources of DPA as a sole end-product; however, after UV irradiation of *Aurantiochytrium* sp., EPA was absent, with increased production of both DPA and DHA [82].

Microalgae-derived omega-3 PUFA can be used in many food-based applications such as infant formulae, nutraceuticals, dietary supplements, pharmaceuticals, foods and beverages [83], and so these products could be tested through clinical trials. However, such clinical trials are relatively rare and have usually been undertaken in healthy individuals (Table 4). While there are many studies on microalgae-derived omega-3 PUFA in diabetic animals, only six small trials have been reported in humans, all on *Arthrospira* rather than omega-3 PUFA producers such as *Nannochloropsis* or the thraustochytrids [47]. In separate trials (Table 4), 19 healthy individuals aged 60–90 years given 2 weeks’ treatment with *Phaeodactylum tricornutum* (Temminck & Schlegel) De Toni containing EPA, carotenoids, vitamins and β-glucans showed anti-inflammatory and potentially antioxidative responses, which could support healthy ageing [50]. *Nannochloropsis*-derived EPA given to 120 healthy adults for 3 months increased the omega-3 index and erythrocyte EPA and DPA concentrations and decreased total and very low-density lipoprotein-cholesterol [51].

### 3.2. Carbohydrates

Carbohydrates are a major source of energy for humans. Carbohydrates with at least three monomeric units that are resistant to digestive enzymes in the intestine are defined as fibre, further divided into insoluble (does not dissolve in water in the gastrointestinal tract) or soluble (dissolves in water to form a gel-like structure) [84]. Table 3 highlights the role of microalgae in providing dietary fibre to meet recommended intake targets.

Dietary fibre, especially soluble fibre, as a source of prebiotics [85], is associated with a healthy gut microbiota. Prebiotics are compounds, mostly oligosaccharides, which are not hydrolysed or absorbed from the stomach but fermented by intestinal microbiota to form short-chain fatty acids (SCFA) such as lactic, butyric and propionic acids for selected bacteria to use as energy to survive [86]. These SCFA have protective effects throughout the body, such as on the digestive, cardiovascular, immune and central nervous systems [85]. Microalgae are a potential source of prebiotics for humans to produce long-term health benefits as they contain a range of intracellular, structural and extracellular functional polysaccharides including starch in the plastids and structural components of microalgae [87] and glycogen in cyanobacteria. Glycogen in cyanobacteria can be manipulated to increase biomass production [88]. The production and secretion of microalgal exopolysaccharides have now been investigated as these polysaccharides have been proposed as antioxidant, anti-viral, anti-fungal, anti-bacterial, anti-ageing, anti-cancer and immunomodulatory agents [89]. Microalgae have been described as sustainable and eco-friendly sources of polysaccharides for use as prebiotics [90]. As an example, the microalgae *Euglena gracilis* Körnicke contains β-1,3-glucans (paramylon) and so has been proposed as a next-generation prebiotic to be included as a component of healthy foods [91].

### 3.3. Protein

Protein is an essential component of human food for the growth and maintenance of cells and tissues. Dietary protein deficiency is widespread, as shown by a Global Burden of Disease 2019 estimate of around 147 million cases of protein-energy malnutrition worldwide, leading to about 212,000 deaths annually [92]. Table 3 highlights the role of microalgae in providing protein to meet recommended intake targets.

Potential sources of protein that may attenuate this global shortfall include animal sources such as beef, pork and poultry. The OECD-FAO predicts an increase of 14% in meat supply to 374 million tonnes by 2030, with the largest increase in poultry, especially in China; greenhouse gas emissions from meat production will then constitute 54% of total emissions from agriculture [93]. Plants including cereals, legumes, pulses and oilseeds could provide sustainable amounts of protein on a global basis, having a lower environmental footprint than animal proteins [94]. Plant proteins are now gaining traction as ingredients for animal-free foodstuffs such as meat alternatives, beverages, baked products and snack foods [95]. Sustainable increases in production of plant proteins such as soybeans may be achievable with improved knowledge of genetic resources in these plants [96]. Agri-waste may prove to be a sustainable resource through which to produce vegan protein [97]. Increasing protein production from microalgae will then widen the choice of alternatives available to relieve protein deficiency.

Increasing protein intake may have greater benefit for defined populations, such as the increasing ageing population, through improving survival by decreasing chronic disease risk [98]. In 2030, 1 in 6 people will be aged over 60 years (1.4 billion), increasing to 2.1 billion in 2050, 80% of whom will live in low- and middle-income countries [99]. Decreased protein intake has been associated with sarcopenia in older adults [100]; sarcopenia is associated with an approximate doubling of the risk of mortality [101]. The source of this protein may be important: in a large cohort of US health professionals, animal protein increased cardiovascular mortality in contrast to the decrease seen with plant protein [102]. Strategies for increasing protein production as part of a sustainable diet providing food security must consider many issues including production costs, environmental concerns, health issues and consumer acceptance [103,104,105]. Changes will be driven in the production of both existing protein sources (such as cereals, meat and dairy) and in emerging protein sources such as pulses, in vitro meat, insects and algae [104,106]. Their higher protein content and suitable amino acid composition make microalgae an advantageous protein source [107]. Further, the environmental benefits of microalgal biomass production include reduced use of land and water, low carbon dioxide emissions and no impact on water pollution [105,108].

Microalgae may provide a sustainable source of protein for animal feeds for both ruminants and non-ruminants to help address global food protein deficiency while furthering the aim of carbon neutrality [109]. The nutritional benefits of microalgae also extend to pet foods, for example, for cats and dogs [110]. Further, increasing microalgae production for use as an animal feedstock could decrease the progression of climate change and simultaneously improve food security. Controlling greenhouse emissions in the livestock industry could decrease the climate risks resulting from the estimated livestock-related production of 7.1 billion tonnes of carbon dioxide equivalent/year, which is around 10-fold higher than that from plants [111]. One proposed intervention is feeding ruminants with the macroalgae *Asparagopsis* to decrease methane production [112]. Supplementation of *Asparagopsis taxiformis* (Delile) Trevisan de Saint-Léon as 1% of the diet combined with the microalgae *Euglena gracilis* Körnicke as 10 or 25% of the diet synergistically reduced methane emissions by 29.9% and 40% without affecting ruminal fermentation [113]. Similar studies with microalgae alone have not been reported. Other products that may ameliorate climate change include microalgae-derived biofertilisers, biostimulants and biochar, which can be used to foster cropping for livestock and human food and possibly to sequester carbon dioxide in a stable form [86].

### 3.4. Vitamins, Minerals and Pigments

Vitamins and minerals are micronutrients essential for life. They are obtained from the diet as humans and animals cannot synthesise most of these micronutrients. There are important differences between different vitamins; for example, group B vitamins and vitamin C are water-soluble and so cannot be stored after dietary intake, unlike the fat-soluble vitamins A, D, E and K, which are stored in the liver and fatty tissues. Unlike terrestrial plants, microalgae can provide useful amounts of vitamins D and K and some B vitamins such as B_6_, B_9_ and B_12_ to alleviate deficiency states, while vitamins A, C and E are also provided in lesser amounts [30]. Microalgae are potential biofactories for vitamins such as β-carotene (pro-vitamin A), vitamins B, C, E and K [114], so dietary intake may attenuate vitamin deficiency states. However, commercial applications of both media and genetic engineering to increase yields of these vitamins are not yet viable [115].

Chronic disease states resulting from vitamin deficiency are well known; examples include beriberi and Wernicke-Korsakoff syndrome (thiamine deficiency) [116] and scurvy (vitamin C deficiency). The prevalence of vitamin C deficiency varies markedly in adults, from 1.4% in England to 74% in north India [117]. Chronic vitamin A deficiency can cause growth and development deficits in children, loss of vision and an increase in the risk of infection; the age-standardised incidence in 2019 was approximately 7%, with highest values in sub-Saharan Africa (23–25%) [118]. Chronic vitamin D deficiency, especially in the elderly, can lead to hypocalcaemia and hyperparathyroidism, increasing the risk of osteoporosis, falls and bone fractures. Marked vitamin D deficiency, defined as a serum 25-hydroxyvitamin D concentration of less than 30 nmol/L, was reported in 15.7% of population from 81 countries in 2011–2022 [119].

Micronutrients also include minerals, with deficiency states occurring with iron, iodine, calcium, zinc, magnesium, potassium and selenium, mainly due to either decreased dietary intake or poor absorption [120]. Iron deficiency is the most common mineral deficiency, estimated to affect more than 30% of the world’s population or around 2 billion people; inadequate iodine status has also been found in 2 billion people [121]. Micronutrient deficiencies are often associated with poverty; they can be addressed by supplementation, fortification and food-based approaches. Iodine deficiency can be minimised by the use of iodised salt, which was introduced in the 1920s, but iodine deficiency is estimated to affect 35–45% of the world’s population [122]. Another example is the possibility of increasing selenium concentrations in plants used for human foods without increasing the uptake of toxic heavy metals such as cadmium, arsenic and mercury [123]. While no studies in humans on the reversal of mineral deficiency using microalgae have been found, supplemental iron from *Nannochloropsis oceanica* (Kützing) Basso, Croot & M.J. van der Merwe increased blood haemoglobin in moderately anaemic mice [52], and supplementation with selenium-enriched *Spirulina* restored selenium concentrations in the liver, kidney and soleus of rats fed a selenium-deficient diet [49] (Table 4).

Further, marine organisms including microalgae are a source of pigments including carotenoids and phycobiliproteins, which likely have health-promoting properties. Marine organisms including microalgae, archaea, bacteria and yeast are potential sustainable commercial sources of carotenoids [124]. Microalgal carotenoids have reported health benefits as antioxidants and anti-microbial and immunomodulatory compounds, alleviating ageing-related diseases such as cardiovascular diseases and diabetes [125]. Phycobiliproteins are photosynthetic light-harvesting pigments from microalgae with pharmaceutical potential, including anti-tumour, antioxidant, hepatoprotective and neuroprotective activities [126].

Investigations on microalgae products as treatments for dietary insufficiency in PUFA, protein, prebiotics, vitamins and minerals strongly suggest that microalgae can be defined as functional foods, especially for malnutrition. Functional foods are now a common theme in biomedical sciences, with a PubMed search of “functional foods” and “review” retrieving 2133 results for 2023 alone. Many definitions are used, with Temple proposing in 2022 that use of the term be restricted to novel foods that have a possible health-enhancing or disease-preventing role at safe and sufficiently high concentrations [127]. Functional foods that may attenuate metabolic syndrome in humans are produced in tropical and temperate climates, similar to the optimal growing conditions for microalgae, including fruit grown in Australia [128] and the Brazilian Cerrado [129] as well as marine biomass such as seaweeds [130]. Microalgae, as unicellular and photosynthetic microorganisms, can now be defined as a potential functional food and nutraceutical for metabolic syndrome [131]. Extension of these roles—for example, into a complementary intervention for healthy ageing with other macronutrients and micronutrients to decrease low-grade inflammageing—should be considered [132].

## 4. Microalgae in Foods

Since the theme of this review is microalgae used to treat undernutrition, the development of acceptable foods [133] and functional foods [134] with microalgae should be examined. Challenges include resistant cell walls, presence of nucleic acids and toxins, and biomass and metabolite productivity [135]. Microalgae are effective biofactories for fatty acids, proteins, prebiotics, vitamins and minerals with nutritional activities [30,33,106,136,137,138]. These properties underlie the use of microalgal products for food. The indigenous people of Asia, Africa and the Americas have used microalgae such as *Arthrospira* and other cyanobacteria as a food component for more than 1000 years [139,140]. Recently, some fine dining restaurants have introduced microalgae into their menus as a commitment to the ideals of sustainability, ethnicity and authenticity [140]. A recent study established sustainable methods of producing microalgae at home. The role of sustainable design in increasing the adoption of microalgae for food use has been studied using a homemade production system of *Spirulina*; a survey to determine inclination to use microalgae; and workshops and tasting sessions leading to product development to increase public awareness and consumption [141].

Microalgae, such as *Nostoc* and *Spirulina*, have been a recorded part of the human diet even before 1900 [142]. Along with nutritional components and health-benefiting components, microalgae are good sources of structural biopolymers such as proteins and carbohydrates, which can help as texturisers or stabilisers in food products [143]. Tomato puree supplemented with photoautotrophic microalgae has shown improved oxidative stability, to which carotenoids from microalgae may have been the primary contributors [144]. *Tetraselmis* and *Nannochloropsis* biomass have shown great potential for use as innovative functional ingredients in bread and crackers. Use of these microalgae improved the antioxidant capacity and amount of bio-accessible polyphenols [145]. Sensory evaluation of breads and crackers with *Tetraselmis* and *Nannochloropsis* showed promising results along with improved nutritional properties [145]. Additions of 0.5 to 1.0% microalgae to broccoli soup improved consumer acceptance along with higher phenolic content and antioxidant activity [146]. A consumer study in Spain confirmed microalgae as sustainable, environmentally friendly, nutritious, healthy and safe for consumption, while a large group of respondents identified lack of information about microalgae as a major problem in improving microalgae intake [147]. In the past five years, around 4,700 products containing microalgae have been launched in the European market, with 99.5% of these containing *Chlorella vulgaris* Beijerinck or *Arthrospira platensis* (Gomont) G. Hassall [148]. Thus, further research is necessary to identify other microalgae that can be incorporated into food products. The use of microalgal PUFA in the food industry is predominantly within the health food market, which represents 75% of annual microalgal biomass production, including incorporation into products such as cheese and pasta as sensory enhancers and preservatives [137,149].

Global consumption of aquatic foods has increased at an average annual rate of 3.0% since 1961 from an average of 9.9 kg/year in the 1960s to 20.2 kg/year in 2020 [23]. Most of this increase has been produced by aquaculture; thus, the requirement for fish food has also markedly increased. Aquaculture uses around 73% of fish oil produced as food [23]. Microalgae has the potential as a viable alternative to fish meal to sustainably supply growth in aquatic foods as part of global food security because it is a suitable food for aquatic animals with low input costs, low carbon footprint, a rapid growth cycle, wastewater treatment benefits and carbon credits from carbon dioxide conversion [150]. Substituting part of fish meal with microalgae in the farming of shrimps, prawns and fish has resulted in increased growth and survival in some species, with no detrimental effects [150]. Challenges include productivity bottlenecks, heavy metal accumulation and decreased food digestibility because of rigid cell walls [150].

Microalgae can be included as an alternative energy source for pigs and sheep; it may help to improve the quality of their meat products and help to improve poultry egg and meat products for human consumption (for example, by increasing omega-3 PUFA) [151]. Ruminants can also utilise the nutrients in algae, either macroalgae or microalgae, which may be included in their diets as a source of protein and energy, possibly increasing the fibre utilisation of poor-quality hay in feedlots [152]. In addition to foods, an obvious use for microalgae biomass is as a feedstock for nutraceuticals and pharmaceuticals [153]. However, if high-value but low-yield products are extracted from the biomass, there will still be a large amount of waste, which doesn’t align with the principles of a circular economy with zero waste. How can this waste from the biomass then be used? This question calls to mind the concept of microalgae as a commercial biofactory producing multiple high-yield outputs such as nutraceuticals, biofuels, polysaccharides and functional foods, in which all or almost all of the biomass will be used [154].

## 5. Commercialisation of Microalgae

Microalgae are economical, sustainable and renewable primary producers for commercialisation in biopharmaceutical, nutraceutical and renewable energy industries [155,156]. Successful use of microalgae as “sustainable biofactories” requires choice of optimal microalgal strains and growing conditions [157]. Industrial production of microalgal products from *Nannochloropsis oceanica* (Kützing) Basso, Croot & M.J. van der Merwe may guarantee process stability, reliability, product quality, sustainability and economic viability, meaning they could compete as functional foods, feed additives and in high-value applications such as cosmetics [158]. Continuing advancements in biotechnology and engineering (including increased photosynthesis by microalgae [159]) and upstream technology (including improved cultivation modes, the design of photobioreactors and control of environmental factors [160]) could drive rapid industrial growth. However, the regulatory framework for the commercial use of new microalgae-based food products in Europe is seen as a commercialisation barrier as it requires lengthy consultation, authorisation and notification processes as part of the European Food Safety Authority (EFSA) approval process, especially for the Qualified Presumption of Safety (QPS) status [161].

Commercial production of microalgae requires large-scale cultivation and high throughput. Key issues of commercial microalgal production include species selection, cultivation technology, nutrient sources, temperature, lighting and harvesting strategies [162,163,164,165]. The range of media used for cultivation now includes source-separated human urine for nutrient removal and recovery [166]. Production options include suspension cultures such as natural ponds and open raceways and the more recently developed closed reaction systems such as photobioreactors, allowing improved purity of species, better control of culture conditions and better removal of contaminants from wastewater [167]. Further, systems using microalgae immobilised on carriers to produce a biofilm have been developed to improve efficiency of microalgae harvesting for biofuels [168] and wastewater management [169]. Innovation and adaptation for food production are continuing [133,170,171,172] with the aim of decreasing costs while increasing biomass production. While the drivers for these changes have predominantly been efficient production of biofuels and remediation of wastewater, similar techniques will become available for increasing microalgae biomass for nutrition.

The use of microalgae has developed over the last 80 years with three aims: creating a staple food for the world after the Second World War, stopped by the “green revolution”; creating a biofuel when oil prices peaked in the 1970s, which lost relevance when oil prices decreased; and managing industrial wastewater, which may be the one aim which has increased in relevance [173]. More recently, the focus of microalgal development has included disparate fields such as bioenergy and improved health. However, microalgae remain an underexplored and underutilised resource that can rapidly produce high yields of biomass. The annual yields of legumes such as cowpeas or soybeans range from 500 kg/ha under dryland conditions to 10,000 kg/ha (10 tonnes/ha) under optimal conditions using irrigation [174]. In contrast, microalgae grow with a practical productivity of 15–30 g dry weight/m^2^/day [175]. A microalgal pond of 250 m × 16 m, so an area of 4000 m^2^, should produce 18–36 tonnes/year in 300 days of production each year. For a comparable land size for legume production, 1 ha (10,000 m^2^) of microalgal ponds would produce 45–90 tonnes of biomass. This increased biomass production of 4.5–9-fold over optimal legume-growing conditions does not compromise fertile agricultural land or use limited freshwater resources. Additionally, no pesticides are used in microalgal biomass production.

Apart from nutritional aspects, microalgae can remove biological, organic and inorganic pollutants efficiently from municipal, agricultural and industrial wastewater [176,177]. By definition, microalgal-based wastewater treatment produces a very valuable resource, purified water with markedly decreased pollutants. Untreated wastewater depletes clean water supply, with adverse economic impacts on agricultural and manufacturing productivity; economic analysis has shown an increase in local economic water scarcity risk from USD 116 billion in 1995 to USD 380 billion in 2010 [178], so the production of clean water by microalgae has a potentially huge economic advantage. One example of the value of microalgae in providing clean water is their use in mine rehabilitation by cleaning up saline or metal-containing water and producing biomass-derived soil improvers for use in agriculture [179]. The uses of microalgae grown in wastewater have expanded to include removal of micropollutants and bacteria from municipal wastewater [180]; contaminants such as endocrine disruptors and pharmaceuticals from domestic wastewater [181]; industrial waste [182], for example from the brewing [183] and steel [184] industries; and remediation of marine sediments contaminated by petroleum hydrocarbons [185]. Wastewater biomass lipids can be used as a substrate for biofuels, while polyhydroxybutyrates from biomass can be used to produce bioplastics to reduce fossil fuel microplastics as ubiquitous pollutants [186,187]. Microplastics can also be bio-remediated by photosynthetic micro-organisms such as wild-type or bioengineered microalgae together with bacteria, fungi or archaea [188].

Biomass from wastewater treatment can also provide high-value agricultural products from wastewater treatment such as biofertilisers, biostimulants and growth additives [189,190,191], including controlled-release fertilisers [192] and microalgal–bacterial biofertilisers [193]. Biopesticides are an environmentally friendly alternative to synthetic pesticides; an extract of the biomass from *Chlorella thermophila* (Schmidle) D. R. Lee & G. F. Smith inhibited the causative agents of bacterial rice blight [194]. Combinations of microalgae and bacteria are useful for restoring degraded and contaminated soils by increasing available N and P and removing contaminants such as heavy metals and pesticides [195]. A further positive in the increased production of microalgae biomass is its ability to capture both carbon and nitrogen oxides. In theory, microalgae could convert 513 tons of carbon dioxide into 280 tons of dry biomass per hectare per year using about 9% light energy [196]. Theoretically, 100,000 km^2^ microalgae farms would reduce the 2020 global carbon emissions by up to 8% [196]. Simultaneous biofixation of both carbon dioxide and nitrogen oxides has been shown by the microalgae species *Chlorella vulgaris* Beijerinck, *Haematococcus pluvialis* (Flotow) R. A. Saunders and *Scenedesmus subspicatus* Kützing [197]. Advances in medical treatments have been proposed for microalgae, including in patients with rheumatoid arthritis [198] and in hypoxic cancers [199], to rescue neuronal activity in the brain [200] and to improve the healing of diabetic ulcers [201]. These examples of current microalgae studies show their potential to provide technological support and cross-funding to support improved nutritional support and clinical interventions using selected microalgae.

## 6. Conclusions

Microalgae show remarkable diversity with different nutritional compositions; thus, different microalgae may provide selective enhancement of either omega-3 PUFA, dietary fibre, proteins or micronutrients, providing the opportunity to choose. Consequently, microalgae species can be chosen for their potential to address different global nutritional challenges. More complete characterisation of microalgal species is important, as are intervention studies to identify their potential applications as food ingredients. This will make them a viable commercial opportunity for future needs. Undernutrition, which is the insufficient intake of nutrients including lipids (especially omega-3 PUFA), carbohydrates, proteins, vitamins and minerals remains a global public health challenge for children, mothers and the ageing population. Nutritional intervention strategies targeting an increased intake of these nutrients from microalgae through different food sources can provide suitable and feasible alternative sources to relieve undernutrition. Various approaches, such as biotechnological applications, can be used to increase microalgal biomass production. Improvements in production will increase knowledge of microalgae and provide opportunities for businesses to grow microalgae and use them for appropriate applications, including as food ingredients. Thus, microalgae can serve as sustainable sources of nutrients to help reduce impact of undernutrition on the world.

## Figures and Tables

**Table 1:** Nutritional composition of microalgae [32,33,34,35].

Microalgae	Protein (%)	Carbohydrate (%)	Lipids (%)
*Chlorella vulgaris* Beijerinck	51–58	12–17	14–22
*Euglena gracilis* Klebs	39–61	14–18	14–20
*Porphyridium cruentum* (S.F.Gray) Nägeli	28–39	40–57	9–14
*Arthrospira maxima* Setchell & N.L. Gardner	60–71	13–16	6–7
*Spirulina platensis* (Gomont) Geitler	46–63	8–14	4–9
*Tetraselmis suecica* (Kylin) Butcher	34–42	10–17	28
*Tisochrysis lutea* (Droop) Bendif & Probert	20–35	15–25	20–30
*Nannochloropsis* sp.	29–32	9–36	15–18
*Arthrospira platensis* (Gomont) G. Hassall	60–66	18–23	2–7
*Schizochytrium* sp.	12	32	38–71

**Table 2:** Lipid composition of microalgae [32,36].

Microalgae	SFA	MUFA	Omega-3 PUFA	Omega-6 PUFA	EPA	DHA
*Nannochloropsis oceanica* (Kützing) Basso, Croot & M.J. van der Merwe	3.71	2.28	2.39	1.39	2.34	-
*Arthrospira platensis* (Gomont) G. Hassall	2.7	0.5	0.01	2.82		
*Isochrysis galbana* Parke	2.04	1.7	3.73	0.25	1.8	1.32
*Phaeodactylum tricornutum* Bohlin	2.76	2.81	3.72	0.5	2.84	0.02
*Porphyridium cruentum* (S.F.Gray) Nägeli	0.61	0.01	0.61	0.84	0.61	-
*Rhodomonas baltica* Kiesel	1.7	0.4	2.15	1.22	0.44	-

Values are presented as % of total lipid content. SFA, saturated fatty acids; MUFA, monounsaturated fatty acids; PUFA, polyunsaturated fatty acids, EPA, eicosapentaenoic acid; DHA, docosahexaenoic acid.

**Table 3:** Improving intakes of omega-3 PUFA, dietary fibre and protein using microalgae.

Nutrient	Average Intake	Recommended Intake	Amount Needed to Achieve This Intake	Fish Oil Production Required	Current Production	Amount of Microalgae Required to Meet Intakes	Annual Cultivation Requirements to Produce Amounts
Omega-3 PUFA [22,37,38,39,40]	<100 mg/day in 100 countries representing 66.8% of the world’s population	250 mg/day	806 tonnes of omega-3 PUFA/day or 294,000 tonnes/year	-	546,000 tonnes fish oil in 2021—75% used in aquaculture; about 15% used for human consumption	3.2 million tonnes dry weight microalgae	Phototrophic: ^A^ 50,000 ha raceways Heterotrophic: ^B^ 253 m^3^ CSTRs
		500 mg/day	490,000 tonnes/year	1.634 million tonnes of fish oil with 30% EPA + DHA		5.4 million tonnes dry weight microalgae	Phototrophic: ^A^ 84,375 ha raceways Heterotrophic: ^B^ 423 m^3^ CSTRs
		1000–1500 mg/day	451,000 tonnes (for 12 weeks)	-		-	-
Dietary fibre [32,41]	20–25 g/day in men; 15–22 g/day in women	30–35 g/day in men; 25–32 g/day in women	25 million tonnes fibre/year	-	-	250 million tonnes of microalgae dry matter each year	Biorefined fractional contribution of ^A&B^ 4.3%
Protein [32,42,43,44]	-	0.6–0.8 g/kg/day	~154 million tonnes protein/year (for 7 billion people assuming a body weight of 50 kg)	-	~77 million tonnes of crude protein from meat, milk and eggs; ~14 million tonnes crude protein from aquatic animals (in 2018)	120 million tonnes of microalgae dry matter each year	Biorefined fractional contribution of ^A&B^ 9%

^A^: based on raceway production of *Nannochloropsis oculata* (Droop) Hibberd at a productivity of 20 g cell dry weight/m^2^/day for a cultivation period of 320 days per year [45]. ^B^: based on production of Thraustochytrids in continuous stirred-tank reactors (CSTRs) at a productivity of 10 g cell dry weight/m^2^/day, a DHA content of 35% of cell dry weight, and a 320-days-per-year production period [46].

**Table 4:** Health benefits of microalgal nutrients in animal models and clinical trials [47,48,49,50,51,52].

Model Used	Microalgal Species (Phylum)	Dose/Form	Effects Observed
Effect of whole microalgae on diabetes mellitus			
Male Albino rats	*Arthrospira platensis* Gomont (Cyanophyta)	500 mg/kg body weight twice weekly for 2 months	Reduced serum glucose concentrations and increased serum insulin concentrations in diabetic rats
Male Wistar rats or Swiss mice	*Arthrospira platensis* Gomont (Cyanophyta)	25, 50, 100 mg/kg body weight for 5 and 10 days	Reduced serum glucose
Male Sprague Dawley rats	*Dunaliella salina* (Dunal) Teodoresco (Chlorophyta)	150 mg/kg body weight at 72, 64, 48, 40 and 24 h before sacrifice for 3 days	Increased body weight and reduced triglyceride concentrations
Male Wistar rats	*Nannochloropsis gaditana* Lubian (Ochrophyta)	10% freeze-dried powder incorporated into diet for 2 months	Increased body weight and decreased serum glucose concentrations
Male healthy and diabetic Wistar rats	*Nannochloropsis oculata* (Droop) Hibberd (Ochrophyta)	Freeze-dried powder at 0, 10, 20 mg/kg body weight/day for 21 days	Increased body weight and serum insulin concentrations, but decreased serum glucose concentrations
Male Wistar rats	*Nannochloropsis oculata* (Droop) Hibberd (Ochrophyta)	Powder at 0, 10, 20 mg/kg body weight/day for 3 weeks	Increased body weight and decreased serum glucose concentrations
Male Sprague Dawley rats	*Porphoridium cruentum* (C. Agardh) Borzi (Rhodophyta)	600, 1200, 1800 mg/kg body weight/day for 14 days	Increased food intake and pancreatic β-cell numbers and granulation. Increased pancreatic islets area at 1200 mg/kg body weight/day
Obesity			
High-carbohydrate, high-fat diet-fed male Wistar rats	*Nannochloropsis oceanica* Hibberd (Ochrophyta)	5% as freeze-dried, mixed in food	Increased lean mass, decreased fat mass, no change in cardiovascular, liver and metabolic parameters or gut structure, increase in relative abundance of *Oxyphotobacteria* in colon
Selenium (Se) deficiency and antioxidant activities			
Female Wistar rats 3 weeks old	*Limnospira platensis* (Gomont) Nowicka-Krawczyk, Mühlsteinová & Hauer Basionym: *Arthrospira platensis* Gomont Synonym: *Spirulina platensis* Gomont (Cyanophyta)	12-week feed trial SS-group: Sodium selenite 20 µg/kg body weight Spi-group: 3 g *Spirulina* powder/kg body weight SeSP group: Se-enriched *Spirulina* biomass (20 µg Se + 3 g Spirulina powder/kg weight	SS-group: minor weight loss in week 12; increased antioxidant activity of glutathione peroxidase and selenoproteins. SeSP group: restored selenium concentrations, especially in liver, kidney, soleus
Healthy individuals			
19 healthy individuals 60–90 years old	*Phaeodactylum tricornutum* Bohlin (Ochrophyta)	2-week diet trial Biomass A (2.3 g/day): Nutrient-replete cultivated lyophilised microalgal power, containing 312.1 mg omega-3 fatty acids (293.5 mg EPA + DHA) SupB (1.8 g/day): Nutrient-deplete lyophilised cellular microalgal extract, containing 1.2 mg EPA + DHA) Biomass A + SupB (2.3 g of biomass A + 1.8 g of SupB)	Diet Biomass A + SupB: reduced plasma concentrations of interleukin-6; improved mobility parameters (5 s sit to stand test; trend gait speed); reduced omega-6/omega-3 ratio; reduced arachidonic acid/EPA ratio
Effect of microalgal ethanol or water extracts on diabetes mellitus			
High-fat, high-sucrose chow-fed male rats	*Chlorella pyrenoidosa* H. Chick (Chlorophyta) or *Arthrospira platensis* Gomont (Cyanophyta)	150 and 300 mg/kg body weight/day for 8 weeks	Improvement in glucose tolerance
Male Wistar rats	*Arthrospira platensis* Gomont (Cyanophyta)	Extracts at 10, 20, 30 mg/kg body weight/day for 3 weeks	Reduced plasma glucose concentrations
Healthy individuals			
Healthy individuals 25+ years	Ethanol extract of *Nannochloropsis* sp. (Ochrophyta)	1 g Almega^®^PL (Qualitas Health (Houston, TX, USA) under the brand name iWi) daily for 3 months, containing 250 mg EPA and polar lipids, or placebo (1 g soy oil)	Increased omega-3 index, EPA concentrations; decreased very-low-density lipoprotein cholesterol without an increase in low-density lipoprotein cholesterol, leading to reduced total cholesterol
Effect of microalgal fatty acids on diabetes mellitus			
Male diabetic (*db*/*db* mice) and healthy mice (CD1 mice)	Chlorophyceae and Eustigmatophyceae	Freeze-dried EPA/DHA at 1 mg/g body weight or 2% incorporated into chow	Microalgal omega-3 fatty acid improved antioxidant activity in adipose tissue in diabetic mice and in the plasma of healthy mice
Effect of microalgal carbohydrates on diabetes mellitus			
High-glucose, high-fat diet-fed Male Kumming mice	*Chlorella pyrenoidosa* H. Chick (Chlrophyta)	150 and 300 mg/kg body weight/day for 8 weeks	Increased body weight and serum insulin concentrations for both doses and increased glucose uptake at 300 mg/kg/day
Male Sprague Dawley rats	*Porphoridium cruentum* (C. Agardh) Drew & Ross (Rhodophyta)	150, 300, 450 mg/kg body weight/day of extracellular polysaccharides for 14 days	Reduced blood glucose, increased food intake, pancreatic β-cell numbers and granulation, and pancreatic islets area
Effect of microalgal pigments on diabetes mellitus			
Female *db*/*db* mice	Marine microalgae	Astaxanthin at 1 mg/mouse/day for 12 weeks	Increased serum insulin and improvement in glucose tolerance
Effect of microalgal iron on iron deficiency			
Iron-deficient C57BL/6 mice (7 weeks old)	*Nannochloropsis oceanica* Hibberd (Ochrophyta)—Fe	6-week trial with control diet (6 mg Fe/kg + 39 mg Fe/kg (iron extracted from microalgal biomass)) Control diet (6 mg Fe/kg) + 1% inulin + 250 units phytase	39 mg microalgal Fe-enriched diet: elevated blood haemoglobin and 2-fold greater liver non-haem iron concentration; increased mRNA of hepcidin and decreased divalent cation transporter 1, transferrin and transferrin receptor 1
